# Book Reviews

**Published:** 1892-05

**Authors:** 


### *BOOK REVIEWS.*

---

Treatise on Gynecology, Medical and Surgical. By S. Pozzi, M. D. Translated from the French by Brooks H. Wells, M. D. Vol. I. New York. Wm. Wood & Co. 1891.

It is with pleasure that we note the publication in the English language of this magnificent work of Pozzi's. Not that there is any lack of works on gynecology at the present day, for the profession certainly has no ground for complaint in that direction. But there is no work in the English language which is so complete and exhaustive as the one before us. It is a marvel of thorough research and as a work of reference has no peer. At the same time it reflects the result of original thought and careful clinical investigation on the part of the author.

The volume opens with several chapters on general methods of examination and treatment. The next subject discussed is the vexed question of metritis. The author follows the German view of regarding all the chronic hyperplasias of the muscular and mucous structures of the wound as chronic metritis. He confesses, however, that he does this from a pathological standpoint. His treatment differs little from the accepted practice in this country, except that he disparages trachelorrhaphy and advocates amputation of the cervix in its stead.

Fibroma of the uterus is discussed in six chapters, and the subject is treated with an admirable degree of thoroughness. The author regards the treatment of fib[illegible]omata by electricity after Apostoli's method as of doubtful value, except as a means of controlling hemorrhage and relieving pain, thus placing himself in accord with the general consensus of opinion among the best authorities in this country.

The three chapters devoted to cancer of the uterus present a complete *résumé* of the present aspect of this subject as regards pathology and treatment. The author is a strong advocate of vaginal hysterectomy in all cases which admit of complete extirpation of the disease and shows by incontestable statistics the superiority of this operation over high amputation.

In the chapters which follow, the author discusses fully displacements of the womb, and shows a strong tendency toward the view generally held in this country, that uterine displacements are of very little importance in themselves. Yet he cites the various methods of treatment in vogue and figures the usual variety of pessaries. It will be noticed, however, that the cure of complicating conditions is regarded of greater consequence than the correction of the position of the womb.

The remaining chapters of the volume are upon "Prolapse of the Genital Organs," "Inversion of the Uterus," "Malformation of the Cervix," and "Disorders of Menstruation."

The second volume of the work will be looked for with interest, and if it fulfills the promise of the first, the entire work will constitute one of the most valuable of recent additions to gynecological literature.

---

Syphilis in Ancient and Prehistoric Times.—By Dr. F. Buret, Paris. Translated from the French by Dr. A. H. Ohmann-Dumesnil, St. Louis, Missouri.
Philadelphia. F. A. Davis Publishing Co.

This is the first volume of a work which will bear the title "Syphilis To-day and Among the Ancients," by Dr. Buret. We have read this book with more than the usual care bestowed by a book-reviewer, and it has interested us from first to last. The position which the author takes is fully expressed by the famous quotation which he makes from Ricord, "In the beginning God created the heavens, the earth, man, and the venereal diseases." The work represents a vast deal of classical research and investigation. The author has searched diligently both historic and prehistoric records for evidence *in re* syphilis, and we fear that at times he has been two eager to procure the desired testimony. In classical literature all mention of cutaneous "eruption," "papules," "ulcers," "erosions," etc., is interpreted to indicate only one thing—the existence of syphilis. The author sees syphilis in one of its manifold forms everywhere. Thus the plague of *Baal Peor* was syphilis; the plague which the Lord visited upon Pharaoh and his house was syphilis contracted from Sarai, the fair wife of Abram; and thus it was syphilis which produced the "rough and discordant voice" of the Sodomites which Chrysostom speaks of. The author's views are plausible but not convincing, and we may say in the words of Holofernes, "He draweth out the thread of his verbosity finer than the staple of his argument." But the book is exceedingly valuable, an excellent contribution to the history of syphilis, and a strong refutation of that curious theory which supposes that the disease suddenly came into existence about the end of the fifteenth century.

---

LECTURES ON TUMORS. By John B. Hamilton, M. D., LL. D.

TAKING COLD. By F. H. Bosworth, M. D.

INSOMNIA AND HYPNOTICS. By Germain See, M. D., and E. P. Hurd, M. D.

The above three monographs are three small volumes of the "Physician's Leisure Library" series, issued by Geo. S. Davis, of Detroit. The names of the authors alone should be a sufficient guarantee of the character of their respective works. The "Lectures on Tumors" have reached a second and enlarged edition, and constitute an excellent contribution to this subject.

In the last of the above mentioned the physiology of sleep is discussed, and also the causes and treatment of insomnia. Lastly, is a chapter on the hypnotics, opium, the bromides, chloral, sulphonal, paraldehyd, chloralamid, etc.

In "Taking Cold," Dr. Bosworth talks popularly of "colds" in general, how we take them, and how to treat them, and also describes their sequelæ, as laryngitis, tonsillitis, etc.

---

THE RATIONAL CURE OF DEAFNESS AND DISCHARGE FROM THE EAR. By Samuel Sexton, M. D., assisted by Dr. Duane. J. H. Vail & Co., New York.

At the request of Dr. Sexton this little work was prepared for publication by Dr. Duane "with the object of giving information concerning the modern treatment of ear diseases by radical measures to numerous inquirers, especially those whose letters I have not the necessary time at my disposal to answer ; and indeed could not answer satisfactorily within the limits of ordinary correspondence." With the exception of the summary of cases reported as being benefited in chronic deafness and discharge there is not much information gained. To get at the technique of the operation one must necessarily read the original articles by Dr. Sexton himself in the Archives of Otology. The cases reported as benefited by the operation are large and certainly, if all be true, the new field of aural surgery will confer a great blessing to humanity. C. D. R.

---

THE YEAR-BOOK OF TREATMENT, for 1892. Lea Bros. & Co., Philadelphia.

This work, which makes its annual appearance from the press of Lea Brothers, is familiar to our readers and does not need much comment here. It is a record of the progress made year by year in all the departments of medicine and surgery, and all important additions to our knowledge, and advances in our art, have been briefly noted. The oft-mentioned "busy practitioner" will find these *Year-Books of Treatment* to abound with useful information readily accessible.

